# The systematic functional characterisation of Xq28 genes prioritises candidate disease genes

**DOI:** 10.1186/1471-2164-7-29

**Published:** 2006-02-17

**Authors:** Anja Kolb-Kokocinski, Alexander Mehrle, Stephanie Bechtel, Jeremy C Simpson, Petra Kioschis, Stefan Wiemann, Ruth Wellenreuther, Annemarie Poustka

**Affiliations:** 1 Division of Molecular Genome Analysis, German Cancer Research Centre (DKFZ), Im Neuenheimer Feld 580, 69120 Heidelberg, Germany; 2 Cell Biology and Biophysics Programme, EMBL Heidelberg, Meyerhofstrasse 1, 69117 Heidelberg, Germany; 3 Institute of Molecular Biology and Cell Culture Technology, Mannheim University of Applied Sciences, Windeckstrasse 110, 68163 Mannheim, Germany; 4 Embryo Gene Expression Patterns, Wellcome Trust Sanger Institute, Wellcome Trust Genome Campus, Hinxton, Cambridge, CB10 1SA, UK

## Abstract

**Background:**

Well known for its gene density and the large number of mapped diseases, the human sub-chromosomal region Xq28 has long been a focus of genome research. Over 40 of approximately 300 X-linked diseases map to this region, and systematic mapping, transcript identification, and mutation analysis has led to the identification of causative genes for 26 of these diseases, leaving another 17 diseases mapped to Xq28, where the causative gene is still unknown. To expedite disease gene identification, we have initiated the functional characterisation of all known Xq28 genes.

**Results:**

By using a systematic approach, we describe the Xq28 genes by RNA *in situ *hybridisation and Northern blotting of the mouse orthologs, as well as subcellular localisation and data mining of the human genes. We have developed a relational web-accessible database with comprehensive query options integrating all experimental data. Using this database, we matched gene expression patterns with affected tissues for 16 of the 17 remaining Xq28 linked diseases, where the causative gene is unknown.

**Conclusion:**

By using this systematic approach, we have prioritised genes in linkage regions of Xq28-mapped diseases to an amenable number for mutational screens. Our database can be queried by any researcher performing highly specified searches including diseases not listed in OMIM or diseases that might be linked to Xq28 in the future.

## Background

**Figure 1:**
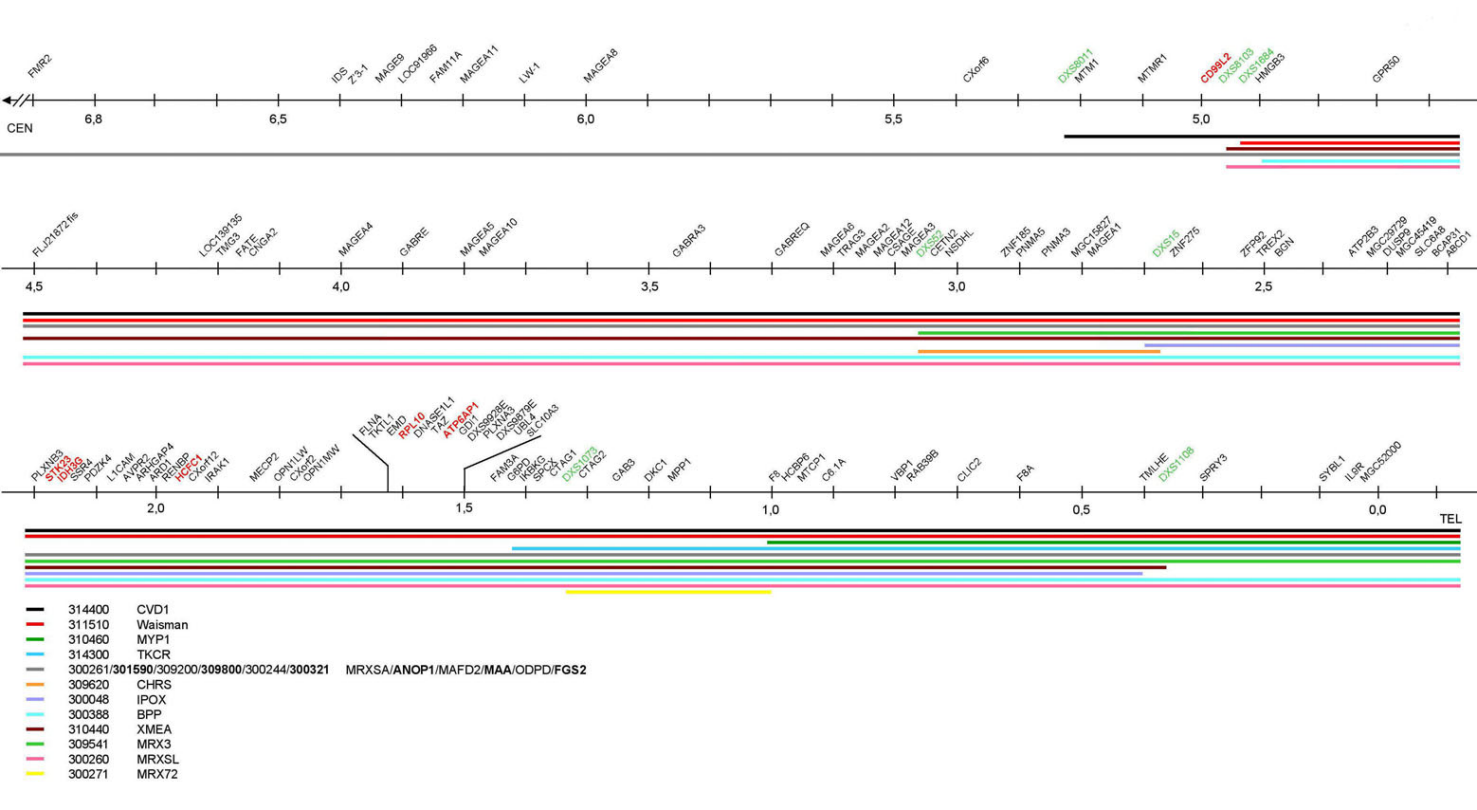
Xq28 map and disease regions. The Xq28 region is shown in a megabase scale and regions of Xq28-mapped diseases are shown as different colored lines. Candidate genes discussed in the text are shown in red. Genetic markers are shown in green.

The human sub-chromosomal region Xq28 has been a focus of genome research for the last 20 years, because many diseases have been linked to this region. Systematic mapping and transcript identification at this region was performed in the early and mid nineties [1-4], while the subsequent availability of the human and mouse genome sequences enabled further gene predictions [5,6]. While gene density along the X chromosome as a whole is below genome average, that of the sub-chromosomal region Xq28 is far above the average (NCBI Map Viewer [7]). Covering approximately 5% of the chromosome, Xq28 harbours almost 13% of the X chromosomal genes. The region spans 7.75 megabases of genomic DNA [8] and harbours 105 non-redundant, confirmed protein-coding genes (NCBI Map Viewer [7]). To date, more than 40 diseases have been mapped to Xq28. For 26 of these, the causative genes have been identified. Among them X-linked adrenoleukodystrophy (*ALD*, OMIM #300100, [9]), X-linked myotubular myopathy (*MTM1*, OMIM #310400, [10]), X-linked Dyskeratosis Congenita (*DKC1*, OMIM #305000, [11]), and Rett Syndrome (*MECP2*, OMIM #312750, [12]). However, for 17 of the Xq28-mapped diseases, the causative gene is still unknown. Ten of them are associated with mental retardation, either as one clinical feature, which is part of a syndrome (syndromic), or as the only primary symptom among affected individuals (non-syndromic). Most of these 17 diseases are rare, limiting fine mapping approaches due to the low patient numbers available for linkage studies. In addition, diagnosis is often imprecise, because of variable phenotypes. Divergence in phenotypes can be caused for instance by environmental factors, the different genetic background of patients, or different mutations within the same gene. The size of the mapped regions of these 17 diseases varies between 0.6 and 7.75 megabases (figure 1) and renders mutational screens for disease association laborious and difficult. For example, the mapped region for the Waisman Syndrome [13] harbours more than 80 genes. To expedite disease gene identification, we took the next step in systematic Xq28 analysis and initiated a functional description of all Xq28 genes. As the tissue expression pattern of a gene particularly at the cell type level, as well as the intracellular localisation of the encoded protein provides highly relevant information regarding function, we systematically studied the Xq28 genes by RNA *in situ *hybridisation, Northern blotting, subcellular localisation, and data mining. Using this functional information, we aimed to narrow down the number of putative disease genes to a manageable set of promising candidates for mutational screens. We organised all experimental data in a relational database enabling comprehensive query options. Using this database with the presumption that all diseases base on single gene mutations, we compared expression patterns of genes with disease phenotypes. This led to the identification of prioritised candidate disease genes for 16 of the 17 Xq28-mapped diseases that do not have a gene associated yet.

## Results

### RNA *in situ *hybridisation

We studied the expression of the respective orthologous mouse genes by RNA *in situ *hybridisation and Northern blot analysis or RT-PCR as the first step of our systematic functional analysis. Fifteen *MAGE-A *or *GABRE *family members were excluded from the total of 105 confirmed human protein-coding Xq28 genes. The human MAGE-A genes are silent in normal tissues, except for male germ cells. And also the mouse orthologs have been described to be not expressed in normal adult tissues, again with the exception of male germ cells [14]. For the remaining 90 genes, 74 mouse orthologs could be identified, all these genes mapping in the synthenic region on the murine X chromosome. For six of the orthologs, the expression patterns have already been published [15-20]. In this work, we analysed tissue expression patterns for the remaining 68 mouse orthologs first by Northern blot or RT-PCR followed by RNA *in situ *hybridisation. Tissue sections of four embryonic stages and ten adult tissues were used to study expression at the cell type level. By combining results from three independent methods, we attained a comprehensive overall picture on the tissue expression profiles of the genes under investigation. We obtained an expression pattern for 65 (94%) of the analysed genes by RNA *in situ *hybridisation and/or Northern blotting/RT-PCR. In Northern blot/RT-PCR analysis, 37 (57%) of the genes showed expression in all tissues analysed. Different splice variants could be detected for 29 (45%) of the genes. In RNA *in situ *hybridisation, 30 genes showed enhanced expression in at least one of the analysed tissues. All tissue expression profiles, as well as original images are accessible through the web-interface of the database, which is described in more detail below. Original images from *in situ *hybridisation were included in the database in all of those cases, where different expression could be observed in different cell types of a tissue, i.e. when *in situ *hybridisation adds more detailed information on expression compared to Northern Blot analysis. *In situ *hybridisation results that are not linked to original images showed an even expression of the respective gene in the different cell types of that tissue. For Northern analysis, all original images are included in the database.

### Subcellular localisation

In parallel, we determined the specific subcellular localisation of the respective human proteins, as the cellular environment contributes information on the possible suite of interaction partners a protein may have and consequently on its potential function(s). Open reading frames of 57 of the human Xq28 genes were amplified and cloned into suitable fluorescent fusion protein expression vectors [21]. Subcellular localisation was analysed by fluorescence microscopy in transiently expressing tissue culture cells. Since the presence of the fluorescence tag can mask important targeting signals present at either ends of the protein, all proteins were expressed and analysed both as N-terminal and C-terminal fusion proteins. Together with original images, the localisations are provided at our web-accessible database.

### Data integration

**Figure 2:**
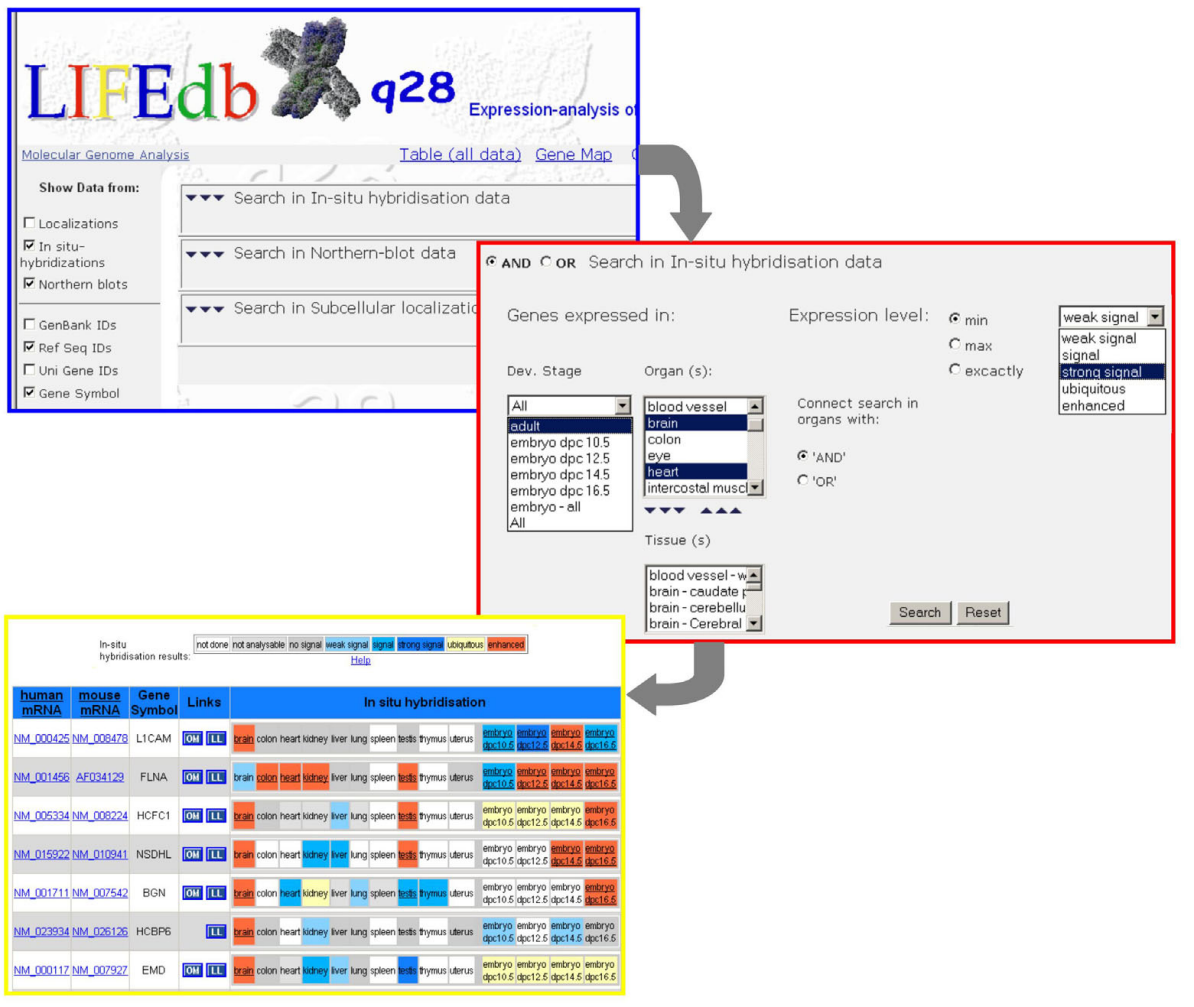
Web-accessible database. The user interface provides comprehensive query options enabling specification of different kinds of experimental data, various organs and tissues as well as signal intensities. The query results are presented in a table with a colour code for signal intensities.

We created an annotation scheme for the analysis of the expression data, differentiating between 29 organs (e.g. brain), 78 tissues (e.g. cerebellum), 40 sub-tissues (e.g. Purkinje cell layer), and 26 cell types (e.g. Purkinje cells). The level of expression was quantified by manual assignment into one of the classes "weak signal", "signal", "strong signal", "ubiquitous", and "enhanced". Together with more than 500 original images and the subcellular localisation results, this information has been organised in a relational database, integrating all experimental results, in addition to web-accessible annotation data for all the genes under investigation. The data can be queried through a web-interface according to various criteria and Boolean combinations ("AND", "OR"), such as developmental stages, tissue types, hybridisation signal intensities, numbers of transcripts, subcellular compartment, or gene identifiers (figure 2). The output matching the selection criteria is either shown in a table together with links to primary data (images, probe and clone information) and to external websites (e.g. NCBI, UCSC) or in XML. In addition to the query interface, a map-like view scaled to chromosomal positions is available, where gene symbols are linked to experimental data, and gene positions to the corresponding regions within the UCSC genome browser. Due to a highly flexible database structure, new data can be entered easily and is readily available via the web-interface [22].

### Identification of candidate disease genes

For the identification of candidate disease genes, we queried the Xq28 database to screen for expression patterns that fit to affected tissues in seventeen Xq28-mapped diseases, for which the causative gene is still unknown. Ten of these diseases are associated with mental retardation, syndromic as well as non-syndromic forms.

First we concentrated on hippocampus, cerebellum, and the olfactory bulb. A number of genes causing mental retardation, for example *MECP2 *(OMIM +300005) [18], NDP (OMIM+310600) [23], *FMR2 *(OMIM +309548) [24], or *SLC6A8 *(OMIM *300036) (this work) have already been reported to have elevated expression in these brain regions compared to other areas of the brain (see table 1). Because of this high compliance we expected that also other genes involved in mental retardation might be highly expressed in these regions. For eighteen genes analysed by RNA *in situ *hybridisation we found an enhanced expression in one or several of these regions (table 1). Four genes, namely *Atp6ap1*, *Hcfc1*, *Idh3g*, and *Cd99l2*, showed enhanced expression in all of the three brain regions.

**Figure 3:**
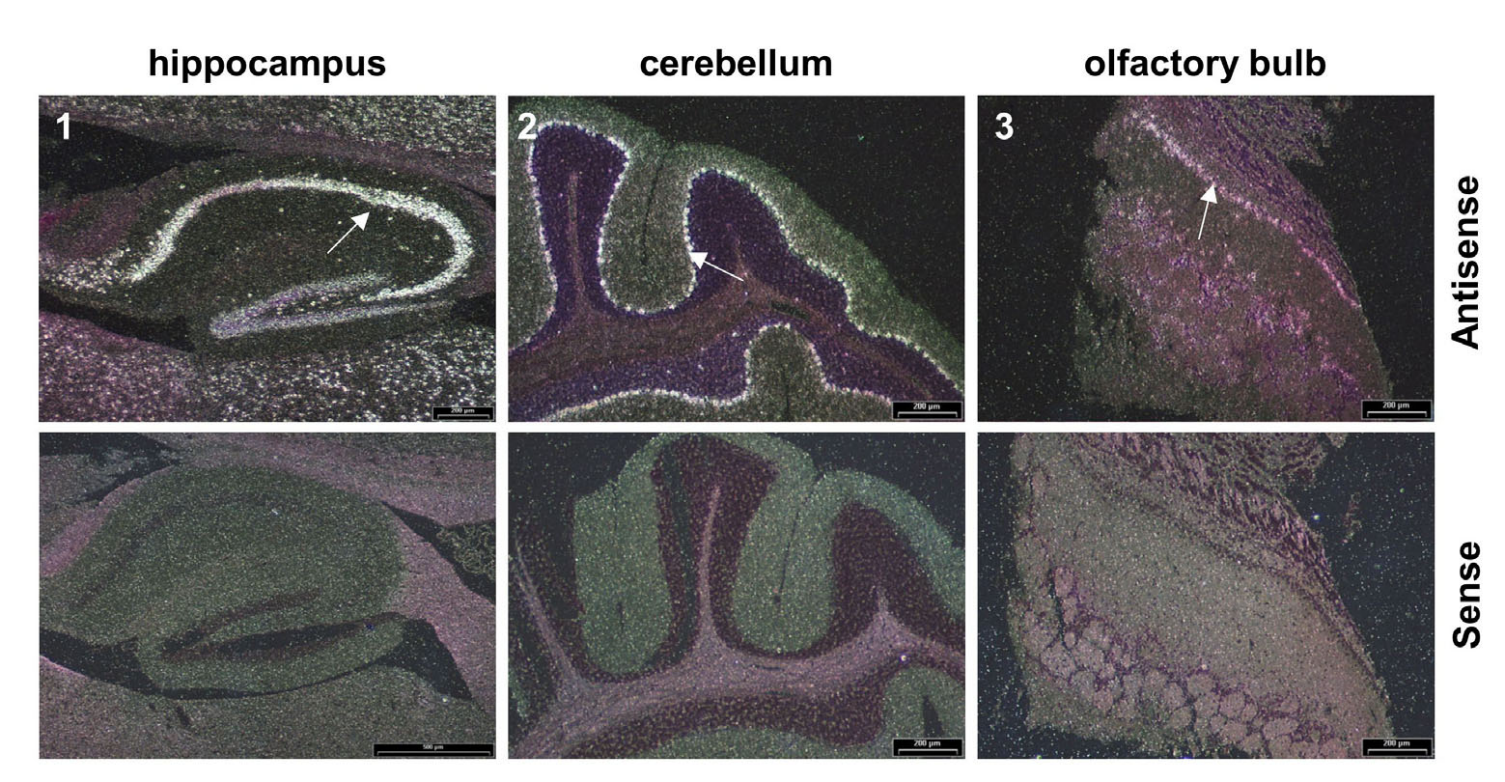
Expression of *Atp6ap1 *in brain. All sections are sagittal and from adult NMRI mice. Upper panel: antisense probe, lower panel: sense probe. 1 hippocampal formation with signal in CA1-CA3 pyramidal cells, 2 cerebellum with signal in purkinje cell layer, 3 olfactory bulb with signal in mitral cells.

In figure 3, expression of *Atp6ap1 *in adult mouse brain is shown. Enhanced expression can be observed in the cells of the hippocampus, the Purkinje cells in the cerebellum and the mitral cells in the olfactory bulb. Results for the other three genes can be viewed at our web-accessible database.

We then extended the analysis to diseases and symptoms other than mental retardation, or diseases where mental retardation is only part of a syndrome. To select tissues for each disease that may be affected, we used the phenotypic description within the OMIM database [25]. Next, we screened our database for genes that show significant expression in tissues affected by the diseases. In this screen, expression patterns of additional genes could be matched to affected tissues of the Xq28-mapped diseases.

We took the mapping information of the 17 Xq28-mapped diseases and listed those genes for each disease, whose expression pattern overlap with potential affected tissues, and which are located in or nearby the respective linkage region. We included nearby genes because linkage analysis often is imprecise. By these means, we prioritised one or several candidate genes for 16 of the 17 Xq28-mapped diseases. The results are summarised in table 2.

**Figure 4:**
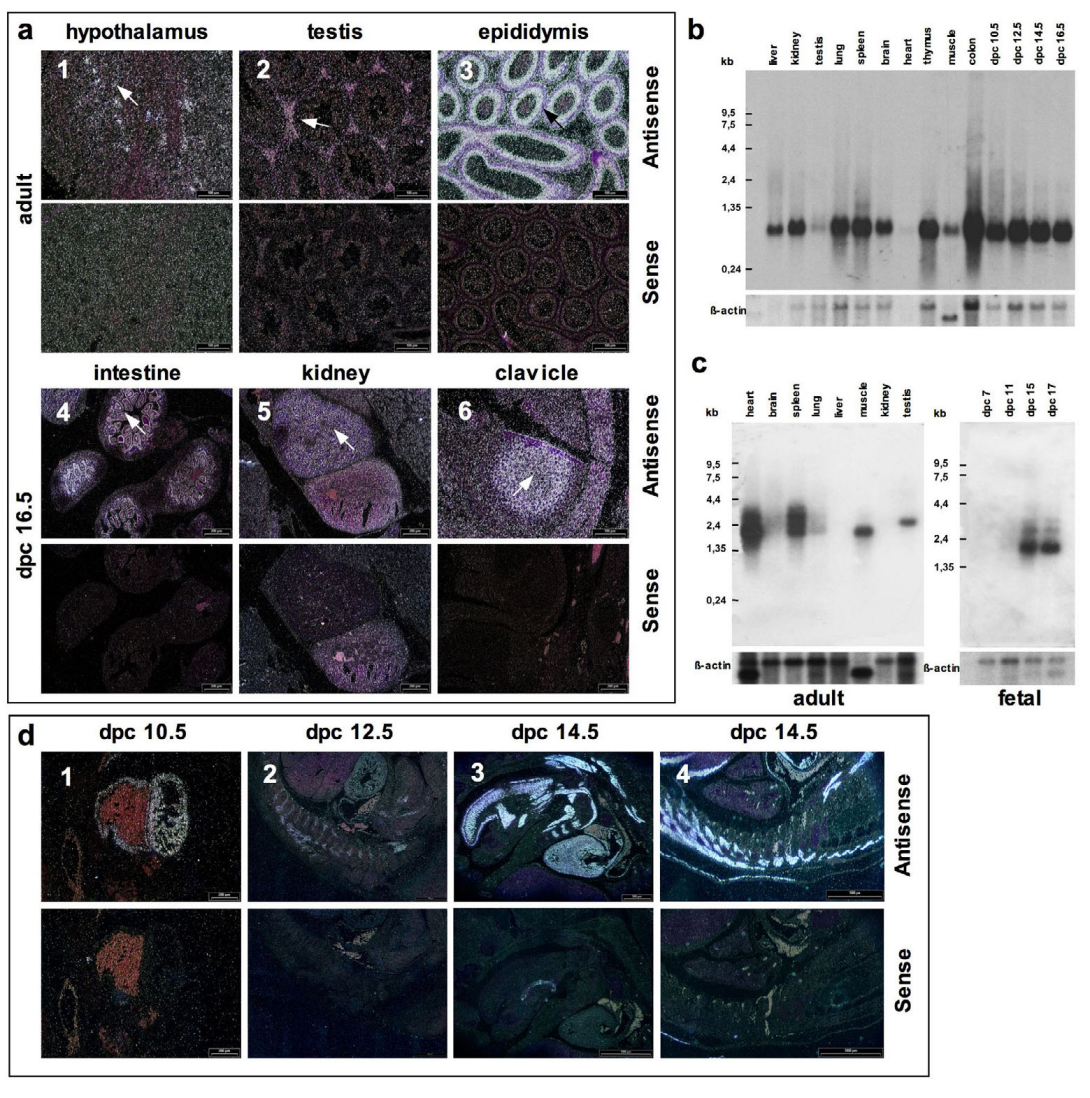
Experimental results for *Rpl10 *(a-b) and *Stk23 *(c-d). **a**. *In situ *hybridisation of *Rpl10 *on adult and embryonic tissues. Upper panels: antisense probe, lower panels: sense probe. 1 hypothalamus with signal in the nucleus of a sagittal brain section, 2 adult testis with signal in leydig cells, 3 epididymis, 4 dpc 16.5 intestine, 5 dpc 16.5 kidney, 6 dpc 16.5 skeletal system/clavicle. **b**. Northern hybridisation with a *Rpl10*-specific probe on a self-made MTN blot, 15 μg of murine total RNA per lane. **c**. Northern hybridisation with a *Stk23*-specific probe on Clontech adult and embryonic MTN blots. **d**. RNA *in situ *hybridisation of *Stk23 *in different embryonic stages. 1 dpc 10.5 heart, 2 dpc 12.5 intercostal muscles, 3 dpc 14.5 tongue and heart, 4 dpc 14.5 intercostal muscles.

Good matches of expression pattern with affected tissues in disease were obtained for *Rpl10 *with Goeminne/TKCR sydrome (OMIM %314300), and for *Stk23 *with X-linked myopathy with excessive autophagy (XMEA, OMIM %310440). *Rpl10 *shows ubiquitous expression in Northern blot analysis (figure 4b), and also in RNA *in situ *hybridisation a signal was obtained in most of the analysed tissues. Strong expression of the gene was observed in the adult brain, especially in the hippocampal formation and cells of the hypothalamus. Also different cell types in the male and female reproductive system have shown a higher expression of the gene, like the leydig cells in adult testis, the pseudostratified columnar epithelium (epididymis) and the cells of the simple columnar epithelium in the cervical glands. The analysis of embryonic tissues has pointed out that the gene is also higher expressed in some tissues of dpc 16.5 embryo, like in the epithelial cells of the gut, in kidney, lung, and thymus as well as in the serous glands in the nasal cavity and the clavicle. Figure 4a presents some of the results from the RNA *in situ *hybridisation. For the serine/threonine kinase 23 gene *Stk23 *the expression was highest in heart, spleen, muscle, and testis by Northern blot analysis, whereas by RNA *in situ *hybridisation, an enhanced signal in embryonic muscle was observed (figure 4c–d).

## Discussion

### Identification of candidate genes for diseases involving mental retardation

The hippocampus, cerebellum, and the olfactory bulb are associated with basic properties of learning and memory in all mammals [26], which is in accordance with the hypothesis that these brain regions are affected tissues in mental retardation syndromes. Mice with a gene knock out of *L1cam *develop a smaller than normal hippocampus with fewer pyramidal and granule cells [27]. Mutations in the human ortholog cause different forms of mental retardation, like the MASA syndrome/spastic paraplegia type 1 (*# *303350) and Hydrocephalus (*# *30700). An enhanced expression in hippocampus, cerebellum, and the olfactory bulb has been shown for several genes known to cause mental retardation, namely *MECP2 *[18], NDP [23], *FMR2 *[24], and *SLC6A8 *(this work), whereas their putative molecular functions are diverse (table 1). Because of these findings, we based our search for candidate mental retardation genes on the hypothesis, that a gene causing a disease is expressed in tissues affected by this disease. So that disease association could be concluded from expression pattern rather than from a particular molecular function. Interestingly, evolutionary conservation of genes causing mental retardation appears to be frequently restricted to vertebrates, as it is the case for five of the six known mental retardation genes shown in table 1. Eighteen genes analysed by RNA *in situ *hybridisation showed an enhanced expression in one or several of these brain regions (table 1). Five of them are known disease genes, but thirteen had not been associated with disease before. The observed expression pattern renders them primary candidates for Xq28-mapped diseases involving mental retardation. Moreover, for five of these 18 genes evolutionary conservation is restricted to vertebrates (table 1). According to their chromosomal location, each of these genes represents a candidate for a subset of Xq28-mapped diseases, for which the disease regions overlap (table 2).

The mouse orthologs of the human genes *ATP6AP1*, *HCFC1*, *IDH3G*, and *CD99L2*, show enhanced expression in all of the three brain regions. The putative molecular functions of these four candidate genes are diverse (SOURCE web tool of Stanford University [28]), as it is the case for the previously described known mental retardation genes (*SLC6A8*, *MECP2*, *NDP*, *FMR2*, and *SLC6A8*, table 1). However, their enhanced expression in hippocampus, cerebellum and the olfactory bulb renders them strong candidates for Xq28-mapped diseases involving mental retardation. Moreover, for two of these four candidates (*ATP6AP1 *and *CD99L2*), phylogenetic conservation is restricted to vertebrates, as can be observed for a number of known mental retardation genes (table 1). For *CD99L2*, a possible association with diseases involving mental retardation could be implicated also by its putative function: Its high expression in neuronal cells is conserved in mammals and zebra fish and suggests a dominant role in neural development [29].

### Identification of candidate genes for other diseases

When searching for candidate genes for diseases other than mental retardation, or for diseases where mental retardation is only part of a syndrome, we stuck to the hypothesis, that disease association correlates with expression in tissues affected by this disease. Based on the hypothesis mentioned above, we searched for expression patterns that match potentially affected tissues of Xq28-mapped diseases, using OMIM phenotypic descriptions to list such tissues [25]. By these means, we could prioritise candidate genes for 16 of the 17 Xq28-mapped diseases (table 2). Between one and 12 genes with matched expression pattern could be listed for each disease, a number which is amenable to mutational screening approaches. Moreover, for most of the diseases, one to four genes with best matching expression patterns could be highlighted (table 2, candidate genes in bold), which could be started within mutational screens. Some of the genes are candidates for more than one disease, because of an overlap of symptoms and the overlap of the candidate regions.

Very good matches of expression pattern with affected tissues could be identified for *Rpl10 *with Goeminne/TKCR syndrome, and for *Stk23*with X-linked Myopathy/XMEA.

TKCR syndrome is characterised by torticollis, keloids, cryptorchidism, reproductive system abnormalities, and renal dysplasia. For *Rpl10*, we observed an enhanced expression in brain, in different cell types of the reproductive system, and in embryonic kidney (figure 4a). The Rpl10 protein is a component of the 60S ribosomal subunit and belongs to the L10 family of ribosomal proteins. It is required for 60S pre-ribosome assembly [30], nuclear export of the 60S subunit [31], and the yeast ortholog Qsr1p was shown to be required for 60S/40S joining [32]. On the X chromosome, *RPL10 *locates very close upstream to the TKCR linkage region, but disease mapping by linkage analysis is often imprecise. Due to its expression pattern, and taking imprecise linkage analysis into consideration, *RPL10 *is a good candidate for TKCR syndrome.

For *Stk23*, a prominent signal in skeletal muscle and heart was observed through all developmental stages. It is also expressed in adult lung, spleen, testis, cerebellum, and the olfactory bulb (figure 4c–d). Due to the remarkable expression pattern in embryonic muscle, *STK23 *could be a candidate for X-linked Myopathy/XMEA. XMEA is characterised by slowly progressing muscle weakness and excessive autophagy. An allelism with Emery-Dreifuss muscular dystrophy has been excluded [33]. *STK23 *is a serine/threonine protein kinase also known as muscle-specific serine kinase 1 (*MSSK1*).

Our hypothesis, that a gene causative for a disease is expressed in tissues affected by this disease, is of course limited, as the timely limited expression of a dysfunctional gene during development for example might lead to a disease, which might become manifest in the adult tissue only. An alternative route to look for disease genes, based on the molecular function of the candidates, could be followed. However, for this route, the molecular functions of the genes within the linkage regions and the molecular pathologies of the diseases have to be known. This is not the case for many Xq28 genes and diseases. Moreover, as mentioned above, for several known mental retardation genes enhanced expression has been shown in brain regions, which are associated with functions impaired in mentally retarded patients.

In this context, our study might help to select genes to start with in mutational screens, where the linkage region of the disease is large and contains a high number of genes.

### Data accessibility

The goal of our study was to use the functional information to prioritise candidate disease genes. For this purpose, it was necessary to organise and integrate this large and heterogeneous dataset. Since our data is organised in a web-accessible database [22], all information may also be used by the scientific community applying highly specified queries with individual focus. By these means, every researcher is able to use the data to prioritise candidate disease genes, both for diseases not listed in OMIM and for diseases that may be discovered and/or mapped to Xq28 in the future. In the last years, a lot of different systematic expression studies have become founded like the approaches on chromosome 21 genes [34,35], as well as the Allen Brain Atlas [36] and the EMAGE gene expression database [37]. All of these projects cover a large amount of genes, but in comparison to our study they present only one tissue type or embryonic stage. With our approach, a large diversity of adult tissues and embryonic stages is covered. Combined with the subcellular localisation data, we gain a lot of information about the genes in this particular region. As the possibility of performing specified queries might also be interesting for similar datasets on other chromosomal regions, researchers are welcome to contact us to get detailed technical information on our system.

## Conclusion

To enhance the speed of gene identification of Xq28-mapped diseases, we have startet to functionally describe the Xq28 genes by expression analysis and subcellular localisation. Our dataset is organised in a relational database with comprehensive query options. This database is freely accessible [22]. We have used the database to match potential affected tissues of Xq28-mapped diseases with expression patterns of genes located within or close to the respective linkage region. By these means, we highlighted candidate genes for 16 of the 17 Xq28-mapped diseases (table 2). By using our database and its query options, every researcher could prioritise candidate genes with individual focus. Our results should enable faster disease gene identification by concentrating on these prioritised candidate genes in mutational screens.

## Methods

### Gene Selection

The NCBI Map Viewer [7] was queried for genes located in Xq28. Novel genes with cDNA/EST representation but without confirmed gene locus were disregarded. Pseudogenes and non-protein coding genes were disregarded, too. The remaining genes were checked for redundancy. Also, the chromosomal localisation of the genes was verified using BLAT search within the GoldenPath human genome browser [38]. This left us with a non-redundant set of 105 human Xq28 genes.

### Selection of orthologous mouse cDNAs

Open reading frames of human Xq28 genes were searched with the BLASTN2 algorithm against different murine databases within the HUSAR program package supplied by the DKFZ Biocomputing Service [39]. The GeneFinder from CGAP [40] and NCBI Map Viewer [7] were cDNA clones suitable for probe generation.

If available, cDNA clones were ordered from the RZPD [41]. Where no cDNA clone was available, or when the cDNA clone was not suitable for probe generation, hybridisation templates were generated by RT-PCR using primers designed on the murine RefSeq entry. All clone and primer information can be retrieved from our Xq28 database [22].

### PCR and RT-PCR

All primer information and PCR conditions for every single clone can be retrieved from our Xq28 database [22].

### RNA *in situ *hybridisation

Manual ISH was performed on embryo sections at stages dpc 10.5, 12.5, 14.5, 16.5 and with different tissues of adult mice. Embryos were isolated from pregnant NMRI mice. The day of plug detection was considered to be day 0.5 postconception (dpc). The tissues and embryonic stages were fixed over night in 4% paraformaldehyde in phosphate-buffered saline at 4°C. The tissues from adult NMRI mice were isolated after perfusion with 4% PFA in phosphate-buffered saline. After embedding in paraffin, 6-μm sections were mounted on 3-aminopropyltriethoxysilane-coated slides and hybridisation was performed as described previously [42] using gene-specific antisense and sense RNA probes. Cloned PCR products were sequence-verified to identify orientation of the product within the vector. The antisense and sense probes were generated by *in vitro *transcription using Sp6 and T7 RNA polymerase, respectively, after linearisation of the construct. Detailed probe information can be retrieved from our web-accessible database. For radioactive hybridisation, α-^35^S-UTP was incorporated into the probes. After washing, the slides were coated with NTB-2 liquid emulsion (IBS Integra Biosciences), exposed for 2 to 4 weeks at 4°C, developed, and counter stained with hematoxylin and eosin. Slides were analysed using an Olympus BX50 microscope. Photographs were taken with a LCD-camera (Power head, Sony) and the AnalySIS software (Soft imaging System GmbH). The figures were assembled using Adobe Photoshop.

### Northern hybridisation

Clontech Northern filters with poly-(A)^+ ^RNA from mouse (7762-1, 7763-1) and self-made RNA- blots with total RNA were hybridised with the ^32^P-labelled purified PCR products also used for cloning into the vector with dual T7/SP6 promoter. Hybridisations were carried out overnight in Church solution (1 M Na_2_HPO_4_, 1 M NaH_2_PO_4_·H_2_O, 10 mM EDTA, pH8.0) at 65°C. Filters were washed once in 0.1% SDS/0.1×SSC for 10 min and once in 0.1% SDS/0.3×SSC for 10 min and exposed to Kodak Bio Max at -80°C.

### Cloning of ORFs into the the Gateway™ cloning system

ORFs were amplified from cDNA clones omitting the 5' and 3' UTRs. PCR primer pairs were selected using the PRIDE program [43]. The 5' end of the forward ORF primer was fixed to the start ATG. To allow expression of C-terminal fusions, the 5' end of the reverse ORF primer was fixed to the last amino acid coding triplet, leaving out the stop codon. Gateway™ recombination sites were attached during PCR by a 2-step strategy: The first PCR was done with gene-specific primers plus a 9 (forward primer) or 11 (reverse primer) base pair overhang. Recombination sites were completed in a nested PCR using the corresponding overhangs. PCR primers were purchased from Invitrogen. Amplification of ORFs was done using the Expand High Fidelity amplification system (Roche). PCR products were cloned by recombination into the entry vector pDONR201 and shuttled into N-terminal CFP and C-terminal YFP expression vectors after sequence validation.

### Subcellular localisation of N- and C-terminal fusions proteins

Localisation analysis has been described elsewhere [21]. In brief, expression plasmids were transfected into Vero cells (ATCC CCL81) using FuGENE6 transfection reagent (Roche). Living cells were imaged at 20 and 40 h after transfection and image acquisition was performed on a Zeiss Axiovert 200 microscope with standard filter sets. All ORFs were analysed as both N- and as C-terminal fusion proteins to minimise the effects of aberrant localisation due to the position of the tag.

### Database design and data processing

Data from construction of expression-clones and protein localisation was processed as described [44]. Probe and clone information and images from *in-situ *hybridisation and Northern blots were stored on a MS-SQL-Server (Microsoft). Data entry was done using MS Access forms as a front-end. For data presentation, a MS IIS-webserver employing the MS.Net framework was used.

## Authors' contributions

AK-K was involved in study design, selected the mouse homologous genes, designed and prepared the probes, performed the RNA *in situ *hybridisation and Northern blot hybridisation as well as the data analysis and the search for candidate genes. AM generated the web-accessible database. SB cloned the open reading frames of the human orthologs. JCS carried out the intracellular localisation of the proteins. PK contributed to the Xq28 map and to study design. SW participated in the Gateway cloning, intracellular localisation of proteins and critical revision of the manuscript. RW has been involved in drafting the article and revising it critically for important intellectual content. AP designed the study, contributed to the drafting and critical revision of the manuscript. All authors have read and approved the final the version of the manuscript.

## Figures and Tables

**Table 1:** Identified candidate genes for diseases involving mental retardation.

**Gene**	**cb**	**hc**	**ob**	**pc**	**Intracellular localisation**	**Hs**	**Mm**	**Rn**	**Xt**	**Gg**	**Dr**	**Fr**	**Dm**	**Ce**	**Sc**	**Ag**	**Pf**	**Potential function**
**known mental retardation genes**																		

*SLC6A8*	X	X	X		n.a.	X	X	X	X		X	X						creatin transporter
*MECP2*	X	X	X		nucleus	X	X	X	X		X	X						methyl CpG-binding protein, regulator of gene expression
*GDI*	X	X	X		cytoplasm	X	X	X	X		X	X	X	X	X	X		RabGDP-dissociation inhibitor involved in synaptic vesicle fusion, neuronal morphogenesis
*NDP*	X	X	X		n.a.	X	X	X		X	X							growth factor
*FMR2*	X	X			n.a.	X	X	X	X	X	X							
*L1CAM*		X	X		n.a.	X	X	X	X	X								integral membrane glycoprotein

**novel candidates for mental retardation**																		

*ATP6AP1*	X	X	X	X	endoplasmic reticulum	X	X	X	X	X	X	X						subunit of a vacuolar H-ATPase
*HCFC1*	X	X	X		nucleus*	X	X		X		X	X	X			X		transcription factor
*IDH3G*	X	X	X		mitochondria	X	X	X	X		X	X	X	X	X	X		NAD(+)-dependent isocitrate dehydrogenase
*CD99L2*	X	X	X		centrosomes	X	X	X	X		X	X						unknown function
*FAM11A*	X				endoplasmic reticulum	X	X	X	X	X	X			X				unknown function
*HCBP6*	X				mitochondria	X	X											unknown function
*BCAP31*	X				endoplasmic reticulum	X	X	X			X		X	X	X	X		regulator of the turnover of endoplasmic reticulum-associated protein tyrosine phosphatase-like B (Yeast)
*IRAK1*		X		X	n.a.	X	X		X			X	X	X		X		serine/threonine kinase
*RPL10*		X			nucleus-nucleolus	X	X	X	X		X	X	X	X	X	X	X	ribosomal protein
*PDZK4*		X	X		nucleus-nucleolus	X	X		X									unknown function
*STK23*	X		X		cytoplasm & nucleus aggregates	X	X	X	X				X	X		X		serine/threonine kinase
*BGN*			X	X	n.a.	X	X	X	X		X	X						connective tissue metabolism by binding to collagen fibrils and transferring growth factor-beta
*CETN2*				X	cytoplasm & nucleus	X	X	X	X	X	X	X				X		calcium-binding protein, structural component of the centrosome

The upper six rows of the table show brain expression patterns, subcellular localisation, evolutionary conservation, and potential molecular function of known mental retardation genes. All other rows list the respective information of a subset of analysed genes found to be expressed in brain. Boxes marked with "X" represent enhanced expression in the respective region (columns 3–6) or existence of an ortholog in the listed species (columns 8–19). Orthologs in other species have been queried from NCBI HomoloGene [45] and Ensemble [46] genome browser. cb: cerebellum, hc: Hippocampus, ob: olfactory bulb, pc: plexus choroideus, Hs: Homo sapiens, Mm: Mus musculus, Rn: Rattus norvegicus, Xt: Xenopus tropicalis, Gg: Gallus gallus, Dr: Danio rerio, Fr: fugu rubripes, Dm: Drosophila melanogaster, Ce: Caenorhabditis elegans, Sc: Saccharomyces cerevisiae, Ag: Anopheles gambiae, Pf: Plasmodium falciparum.

**Table 2:** Xq28-mapped diseases and favoured candidate genes.

**OMIM ID**	**Disease linked to Xq28**	**Candidate region**	**Potential affected tissues**	**Candidate genes**
311510	PARKINSONISM, EARLY-ONSET, WITH MENTAL RETARDATION; WAISMAN SYNDROME (WSN)	DXS1684-Xqter	brain: substantia nigra, basal ganglia, white matter, frontal cortex, adrenal glands	***ATP6AP1***, *STK23, NSDHL*, ***HCFC1***, *RPL10, PDZK4*, ***SLC6A8***, ***IDH3G***, *BCAP31, IRAK1, HCBP6, IKBKG*
314400	CARDIAC VALVULAR DYSPLASIA, X-LINKED (CVD1)	DXS8011-Xqter	heart: myocardium, valves, joints, cartilage	***STK23***
309541	MENTAL RETARDATION, X-LINKED 3 (MRX3)	DXS52-Xqter	brain	***HCFC1***, ***SLC6A8***, ***ATP6AP1***, ***IDH3G***, *BCAP31, STK23, PDZK4, IRAK1, RPL10*, ***IKBKG***, *HCBP6*
310440	MYOPATHY, X-LINKED, WITH EXCESSIVE AUTOPHAGY (XMEA)	DXS8103-DXS1108	peripheral nervous system, muscle	***STK23***
314300	TORTICOLLIS, KELOIDS, CRYPTORCHIDISM, AND RENAL DYSPLASIA (TKCR)	G6PD-Xqter	brain, testis, embryo: kidney	*ATP6AP1, NSDHL*, ***RPL10***, *FAM3A*
310460	MYOPIA 1 (MYP1)	F8-Xqter	brain: midbrain, brain stem, eye: retina, lens	*NSDHL, BGN*
300261	ARMFIELD X-LINKED MENTAL RETARDATION SYNDROME (MRXSA)	Xq28	brain, embryo: skeleton	*IDH3G*, ***RPL10***, *SSR4, PDZK4*
300260	LUBS X-LINKED MENTAL RETARDATION SYNDROME (MRXSL)	DXS8103- 5CM	brain, muscle	*STK23*
309620	MENTAL RETARDATION, SKELETAL DYSPLASIA, AND ABDUCENS PALSY; CHRISTIAN SYNDROME (CHRS)	DXS52-DXS15	brain, joints, cartilage, spinal cord, embryo: skeleton, pancreas, liver	*NSDHL*
300048	INTESTINAL PSEUDOOBSTRUCTION, NEURONAL, CHRONIC IDIOPATHIC, X-LINKED (IPOX)	DXS15-DXS1108	intestinal tract: innervation, wall, colon, blood, embryo	***RPL10, FLNA, RENBP***, *ATP6AP1,*
300388	POLYMICROGYRIA, BILATERAL PERISYLVIAN (BPP)	DXS8103-Xqter	brain: hippocampus, midbrain, brain stem, temporal cortex, parietal cortex	*BCAP31*, ***IDH3G***, *HCFC1, IKBKG, SLC6A8, PDZK4,*
300321	FG SYNDROME 2 (FGS2)	Xq28	brain: white matter, motor cortex, stem ganglia, corpus callosum, central and peripheral nervous system, embryo: brain	***IDH3G***, ***CD99L2***, *FAM11A,*
300244	TERMINAL OSSEOUS DYSPLASIA AND PIGMENTARY DEFECTS (ODPD)	Xq27.3-q28	bone, eye, skin,	***SSR4*, ***RPL10*, ***BGN***
300271	MENTAL RETARDATION, X-LINKED 72 (MRX72)	DXS1073-F8c	brain	no gene with matched expression pattern is located within or near linkage area
301590	ANOPHTHALMOS, CLINICAL (ANOP1)	Xq27-q28	bony orbits, brain, skin	*HCFC1, ATP6AP1, CD99L2, IDH3g, PDZK4, FAM11A*
309200	MAJOR AFFECTIVE DISORDER 2 (MAFD2)	Xq28	brain	***HCFC1***, *SLC6A8*, ***ATP6AP1***, ***CD99L2***, *IDH3G, PDZK4, FAM11A*
309800	MICROPHTHALMIA WITH ASSOCIATED ANOMALIES (MAA)	Xq27-q28	eye, bone, urogenital, heart, teeth	*SSR4*, ***RPL10***, *IDH3g*, *STK23*

Columns one and two list those Xq28-mapped diseases, for which the causative gene has not yet been identified. Flanking genetic markers of disease regions are shown in the third column. Potentially affected tissues (column four) have been selected according to phenotypic descriptions within the OMIM database. Column five lists candidate genes that have been identified by matching affected tissues with RNA *in situ *hybridisation patterns using the query options of our web-accessible database, and combining with information on the chromosomal location of the genes and diseases. Genes with best matches are shown in bold.
